# The Antiapoptosis Effect of* Geum japonicum* Thunb. var.* chinense* Extracts on Cerebral Ischemia Reperfusion Injury via PI3K/Akt Pathway

**DOI:** 10.1155/2018/7290170

**Published:** 2018-11-14

**Authors:** Bingjin Ou, Wei Tao, Songbai Yang, Jiateng Feng, Jinfeng Wang, Tong Yang, Hanyu Wu, Yaguang Huang, Lingjing Tan, Weifeng Huang, Zhitao Feng, Zhigang Mei

**Affiliations:** ^1^Third-Grade Pharmacological Laboratory on Chinese Medicine Approved by State Administration of Traditional Chinese Medicine, Medical College of China Three Gorges University, Yichang, Hubei 443002, China; ^2^Yichang Hospital of Traditional Chinese Medicine Affiliated to China Three Gorges University, Yichang, Hubei 443003, China

## Abstract

*Geum japonicum *Thunb. var.* chinense *(GJ) is a type of wild vegetable found in China and other Asian countries; it has been reported that its extracts possess a neuroprotective effect against cerebral ischemia reperfusion (CIR) injury. The aim of this study is to explore the effect GJ extracts on transient focal CIR injury and neurons apoptosis and to clarify its possible underlying mechanisms* in vivo*. Our results indicated that pretreatment with GJ extracts significantly ameliorated the infarct volume, decreased neurological deficits, lessened neural cells apoptosis, downregulated GFAP activity level, and increased surviving neurons. Moreover, GJ extracts preadministration increased Bcl-2 levels and attenuated the increase in the expressions of Bax and it also lowered the cleaved caspase-3 activity in ischemic cortex tissues which was caused by CIR and increased the expression of PI3K and p-Akt. The above effects of high dose of GJ (GJ-H) group were much better than those of low dose of GJ (GJ-L), which indicated that GJ extracts may be helpful in the suppression of CIR injury with a dose-dependent manner.

## 1. Introduction

Stroke, a life-threatening disease, is the second most common cause of death worldwide, and a major healthcare problem in Asian [1]. There are 10.3 million or so new cases of stroke were diagnosed in 2013, and an estimated 6.5 million people have died of stroke during the year [2]. About two-thirds of strokes occurred in low- and middle-income countries, especially in South, East, and South-East Asia [3]. Ischemic stroke, which is caused by the blockade of cerebral blood flow, is the most common type of stroke, accounting for 60 to 80 percent among all stroke cases [4]. A permanent or transient decrease in cerebral blood flow frequently causes cerebral ischemia, leading to the damage of nerve in the brain [5]. To restore blood flow is the principal way to treat ischemic stroke clinically, fibrinolytic therapy by recombinant tissue plasminogen activator (rt-PA), the only thrombolytic drug approved by the US Food and Drug Administration in 1996, is still the most efficient treatment for ischemic stroke [6], but only few percentage of patients are eligible for rt-PA therapy, because of its secondary reperfusion injury [7, 8].

Cerebral ischemia reperfusion (CIR) results in a series of pathological reaction including inflammation, calcium overload, glutamate excitotoxicity, oxidative stress, apoptosis, and so on, which may cause delayed neuronal and astrocytic death [9–11]. Neurotransmitter turnover, ion homeostasis, and other important processes can be controlled by functional interactions between neurons and astrocytes, and these physiological processes require uninterrupted energy supply; energy depletion of astrocytes caused by hypoxia-ischemia will disrupt signaling and ionic gradients, severely impair brain function, and lead to cell death [12, 13]. In some studies, astrocytes undergo dysfunction and death prior to neuronal death after focal or global cerebral ischemia [14]. Evidence has demonstrated that apoptosis after CIR is one of the major contributors to the process of cell death [15, 16]. In addition, the apoptosis process induced by ischemia could be facilitated and accelerated by reperfusion [17]. Therefore, the survival of neurons and astrocytes under CIR conditions is important for facilitating ischemic stroke recovery.

The whole plant of* Geum japonicum* Thunb. var.* chinense* (GJ, Lanbuzheng in Chinese), which is increasingly being used as functional vegetables or a kind of folk medicine in China or other Asian countries, has been used for the alleviation of dizziness and headache by some Chinese or other Asian [18]. Furthermore, GJ, as a main component of some Chinese herbal formulas, has been found helpful for the recovery after stroke, with improved functional ability and independence in activities of daily living for patients [19, 20]. However, its mechanism of action has not been fully clarified. In recent years, Liu et al. [21] indicated that GJ extracts could increase the expression level of Bcl-2, inhibit the activity of Bax, improve the ability of brain tissue anti-ischemic injury, and alleviate apoptosis death of neurons. Accumulated evidences suggested that stabilization of the ratio of Bcl-2 and Bax protein levels played a leading role in regulating apoptotic cell death. Increased expression of Bcl-2 inhibits apoptosis, while overexpression of Bax involves the promotion of apoptosis [22, 23]. Moreover, the phosphatidylinositol 3-kinase (PI3K)/Akt signaling pathway is generally thought to play an important role in promoting cell survival and inhibiting cell apoptosis after brain ischemia. So, it may be an important therapeutic target for preventing cellular death induced by cerebral ischemia [24, 25]. Hence, in the present study we are planning to investigate whether GJ extracts exhibit the beneficial effects against apoptosis following CIR injury via the PI3K/Akt pathway in the CIR model rats.

## 2. Materials and Methods

### 2.1. Herbal Preparation

*Geum japonicum* Thunb. var.* chinense* (GJ), belonging to Geum in Rosaceae family, has been recorded in the 2015 edition of the Pharmacopoeia of the People's Republic of China. In our present study, the herb was purchased from the Anhui Sanyitang Pharmaceutical Co., Ltd., Anhui province, China. It was harvested in Guizhou province of China and was identified by Dr. Yumin He and Professor Jianhong Zeng, Medical College of China Three Gorges University, Hubei, China. A voucher specimen was deposited (dry conditions, normal temperature) in the Medical College of China Three Gorges University, Hubei, China. The dried plant material was extracted three times with 70% ethanol (1:10) in flask heating mantles at 60°C, 1.5-2 hours each time and then filtered through a two-layer filter screen and collected the residue concentrated in a boiling water bath. GJ extracts were stored in 4°C before experiment. We chose two doses of GJ extracts: high dose of GJ (GJ-H, 7.00g/kg) and low dose of GJ (GJ-L, 1.75g/kg) in the present study, and the dose selection of the extracts is mainly based on Liu et al. [21, 26, 27] and our clinical dosage of GJ for the treatment of headache and ischemic stroke.

### 2.2. Experiment Animals

The seventy-two SD rats (weighing 220-250g, 6-8 weeks old, SCXK2017-0012) were purchased by the Animal Center of China Three Gorges University (Yichang, China) for preparing the model of middle cerebral artery occlusion (MCAO). All experiments were approved by the Ethics Committee of China Three Gorges University, and in accord with the guidelines of the National Institutes of Health on the care and use of animals.

### 2.3. The Procedures of MCAO

After finishing the administration with GJ extracts, focal cerebral ischemia was induced by using an intraluminal filament technique as described previously [28]. Briefly, rats were injected intraperitoneally with 10% choral hydrate (Sinopharm Chemical Reagent Co., Ltd., Shanghai, China) at the dose of 3.5 ml/kg. Through a ventral midline neck incision, all the right common carotid artery (CCA), external carotid artery (ECA) and internal carotid artery (ICA) were exposed. After a small, a nylon monofilament (Cinontech Co., Ltd., Beijing, China) about 40 mm in length and 0.24-0.26 mm in diameter, was transiently introduced into the lumen of the ICA from the incision snipped open at 5 mm behind the bifurcation. The monofilament was advanced 18-20 mm long past the carotid bifurcation into the ICA lumen while feeling the mild resistance and then fastened the blood vessel and sutured the neck skin. After 90 min of occlusion, this inserted filament was carefully removed to restore the blood flow. The sham-operated group rats underwent the same experimental procedures but without the incision on CCA and the insertion of the filament. After finishing this procedure, the rats were kept in the same environment as before the operation.

### 2.4. Animal Grouping and Treatment

The seventy-two male SD rats were divided equally into four groups, including the sham, I/R, GJ-H: (7.00g/kg GJ +I/R), GJ-L: (1.75g/kg GJ +I/R). GJ extracts were gavaged to rats route once daily for 7 days before MCAO. An animal's ability, comprised of drinking, feeding, and ambulation as well as its general appearance was assessed after gavage. All the rats except for the sham group were induced CIR injury by undergoing MCAO surgery; the middle cerebral artery was occluded using intraluminal filament and restore blood flow after 1.5 h. In accordance with the requirement of Experimental Animal Management Committee of China Three Gorges University, the animal must be humanely killed by CO_2_ inhalation to prevent animal suffering if the animal became severely ill in experimental process.

### 2.5. Neurological Evaluation

In accordance with Zea Longa [29] scoring, neurological deficits were evaluated on a five-point scale system after 24 h of reperfusion by an observer blinded to the animal grouping system, the specific symptoms are graded with minor modifications as previously described [25], and a score over 3 points or dead rats were eliminated.

### 2.6. TTC Staining

After behavioral evaluation, rats were anesthetized and decapitated; the brain tissues were removed rapidly from the skull and then sliced into five 2-mm-thick coronal slices at 2 mm distance from forehead after freezing 20 min at −20°C. A ratio of one tissue to five sections was employed. The tissue slices were stained with 2% TTC at 37°C for 30 min and then fixed in 4% paraformaldehyde overnight. Infarct size was analyzed using Image-pro plus 6.0 (Media Cybernetics, Maryland, MD, USA) who were blinded to the experimental group. In order to exclude the effect of cerebral edema, the infarct size was standardized to the nonischemic hemisphere and expressed as percentage of the contralateral hemisphere.

### 2.7. Immunofluorescence Staining

GFAP in the ischemic cortical area was examined by immunofluorescence after 24 h of reperfusion. Animals were anesthetized and the brain tissues were perfused with 4% paraformaldehyde from cardiac apex; the separated brains were embedded in paraffin according to procedures carried out as previously described [30]. Paraffin sections were fixed with 4% paraformaldehyde for 12 min and 0.25% Triton for 5 min and blocked in 1% BSA (Servicebio, China) diluted in PBS at room temperature for 1 h and then incubated with primary antibody anti-GFAP (dilution of 1:1000, Google bio Co., Ltd., China) at 4°C overnight. After rinsing with PBST, sections were administrated with Cy3-conjugated anti-rabbit IgG (dilution of 1:300, Google bio Co., Ltd., China) for 1.5 h at room temperature, next incubated with DAPI (Google bio Co., Ltd., China) for 10 min. The brain sections were coverslipped, imaged and photographed by fluorescence microscope (Nikon, Japan). Finally, the stained cells were counted using Image-Pro Plus software by an investigator who do not know experimental design. Each experimental group contained at least five brain sections for staining examinations.

### 2.8. Nissl Staining

Histologic sections of the right hemisphere underwent Nissl staining after 24 h of reperfusion. The sections produced as above were dewaxed, rehydrated, and immersed in toluidine blue (Servicebio, China) solution for 5 min, and the sections were rinsed with distilled water, dehydrated in ethanol and xylene, and then coverslipped with neutral balsam (Sinopharm Chemical Reagent Co., Ltd., Shanghai, China). Images in the prefrontal cortex were captured using the light microscope (Olympus, Japan). The number of Nissl bodies in cortical area was quantified using Image-Pro plus 6.0.

### 2.9. TUNEL Staining

Neural cells apoptosis was measured suing the terminal deoxynucleotidyl transferase Mediated Nick End Labeling (TUNEL) assay according to the In Situ Apoptosis Detection Kit (Roche, Switzerland). In brief, the paraffin sections were blocked in 3% hydrogen peroxide for 10 min and then incubated with buffer solution at 37°C for 20 min. Sections were added with a terminal deoxynucleotidyl transferase (TdT) reaction mix at 37°C for 2 h, next incubated with Converter-POD solution at 37°C for 40 min and after that colorized with 4′6-diamidino-2-phenylindole (DAPI) (Google bio Co., Ltd., China). For negative control, sections were not incubated with TdT reaction mix. The apoptotic index was presented as percentage of TUNEL-positive cells to the total number of cells under the same field.

### 2.10. Immunohistochemistry Staining

Immunohistochemistry was used to detect the Bcl-2, Bax, and cleaved caspase-3 positive cells in the ischemic cortical area after 24 h of reperfusion. Paraffin sections were dewaxed and hydrated in graded concentration of ethanol and xylene; antigen retrieval was conducted in citric acid (pH 6.0) (Servicebio, China). Sections were incubated with 3% hydrogen peroxide for 10 min to block endogenous peroxidase and added with 1% BSA (Servicebio, China) for 30 min and then mixed with monoclonal antibody (rabbit anti-mouse Bcl-2, dilution of 1:100; rabbit anti-mouse Bax, dilution of 1:200 rabbit anti-mouse cleaved caspase-3, dilution of 1:300. All from Servicebio, China) overnight at 4°C. Next, sections were mixed with polymerase for 20 min and incubated with horseradish peroxidase-conjugated goat anti-rabbit IgG monoclonal antibody (dilution of 1:200, Servicebio, China) for 1 h at room temperature. DAB (Servicebio, China) was used as chromogen and hematoxylin as counterstain after a rinse in distilled water, section dehydrated, and coverslipped. The expression level of Bcl-2, Bax, and cleaved caspase-3 was calculated as number of positive cells/total cells number. The observers who recorded measured results were blind to the experimental design.

### 2.11. Western Blotting

The right cortex was harvested at 24 h reperfusion and stored in a −80°C refrigerator, and the tissue was lysed with RIPA buffer (Beyotime, China) containing 1 mM PMSF (Beyotime, China) after completely grounding. Protein concentrations were quantitated using a BCA Protein Assay reagent kit (Beyotime, China). Loading equal quantities of protein were selected and electrophoresis was performed using 12% polyacrylamide gel, then electrophoretically transferred onto a polyvinylidene difluoride (PVDF) membranes (Millipore, USA), and nonspecific binding was blocked by Tris-buffered saline and Tween 20 (TBST) with 8% milk in for 1 h at room temperature and incubated in a plastic box with the following antibodies overnight at 4°C: rabbit anti-mouse Bcl-2 monoclonal antibody (dilution of 1:800, Abcam, UK), rabbit anti-mouse Bax monoclonal antibody (dilution of 1:6000, Proteintech Group, Inc., China), rabbit anti-mouse Caspase-3 monoclonal antibody (dilution of 1:1000, Proteintech Group, Inc., China), rabbit anti-mouse cleaved caspase-3 monoclonal antibody (dilution of 1:200, Boster Biological Technology Co. Ltd., China), rabbit anti-mouse PI3K monoclonal antibody (dilution of 1:1000 Proteintech Group, Inc., China), rabbit anti-mouse Akt monoclonal antibody (dilution of 1:1000, Cell Signaling, USA), rabbit anti-mouse p-Akt monoclonal antibody (dilution of 1:1000, Cell Signaling, USA), and rabbit anti-mouse *β*-actin monoclonal antibody (dilution of 1:200, Boster Biological Technology Co. Ltd., China). After washing by TBST, the PVDF membranes were incubated with the secondary antibodies (dilution of 1:50000, Boster Biological Technology Co., Ltd., China). Densitometric analysis was performed using BandScan5.0 analysis software. For statistical analyses, all target proteins were quantified using density analysis normalized to *β*actin and then calculated as percentage of control group. Each set of experiments should be repeated three times to correct for deviations.

### 2.12. Statistical Analysis

All data were examined using SPSS 19.0 statistical software. The statistical results were expressed as mean ± standard error of mean (SEM). Comparisons of the multigroup were assessed by the one-way analysis of variance (ANOVA), and two-group comparisons were measured using a* t* test. A value of* p*<0.05 was considered statistically significant.

## 3. Results

### 3.1. Effects of GJ Extracts on Neurological Deficit Scores and Cerebral Infarct Volume

**Figure 1:**
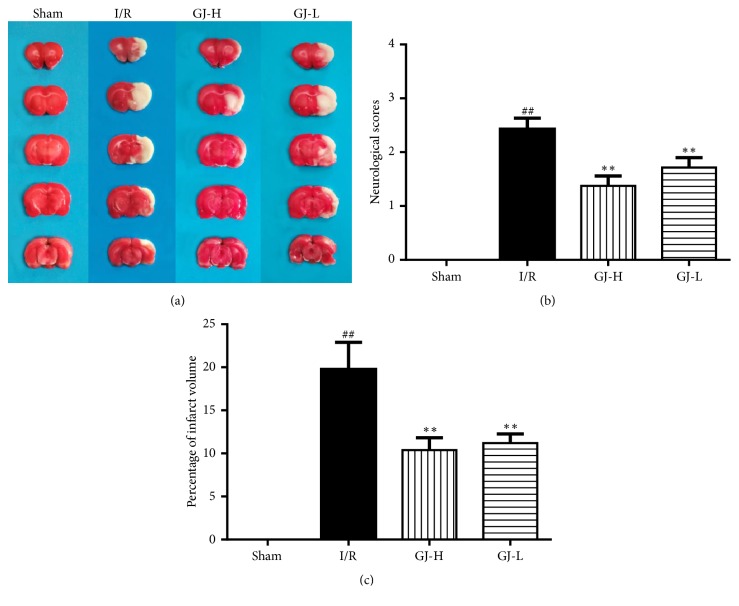
Treatment of different concentration of GJ extracts 7 days prior to MCAO reduced infarct volume and ameliorated neurological deficit in rat 24 h after reperfusion. (a) Representative TTC-stained photos of the cerebral infarct coronal sections. (b) Neurological deficit scores. (c) Percentage of cerebral infarct volume. All data were presented as mean ± SEM. ^##^*p*<0.01 versus sham group; ^*∗∗*^*p*<0.01 versus I/R group, respectively.

Neurological deficit scores system is according to the rat behavior after 24 h reperfusion to evaluate scathing degree; the higher the scores were, the more serious the injury was. As show in Figure 1(b), compared with the sham group with no deficit symptoms, the scores were remarkably raised in I/R group (2.43±0.20) (*p*<0.01). Interestingly, advanced administration with GJ extracts could alleviate the high score in I/R group; the scores of GJ-H groups (1.38±0.18) and GJ-L (1.71±0.18) were significantly decreased in comparison to I/R group (both* p*< 0.01). The subdued movements observed were in a gradual improvement via this scoring system. To investigate the neuroprotective effect of GJ extracts against CIR injury, brain infarct volume was assessed by TTC staining after 24 h reperfusion. The data showed that the percentage of infarct volume in I/R group (19.8±3.1%) was significantly increased (*p*<0.01) compared with the sham group with no infarction. Moreover, pretreatment with GJ extracts was protective and could significantly reverse the brain infarction; the percentage of infarct volume in GJ-H (10.4±1.4%) and GJ-L (11.2±1.1%) group was significantly decreased compared to the I/R group (both* p*<0.01) (Figure 1(c)).

### 3.2. Effect of GJ Extracts on GFAP Expression

**Figure 2:**
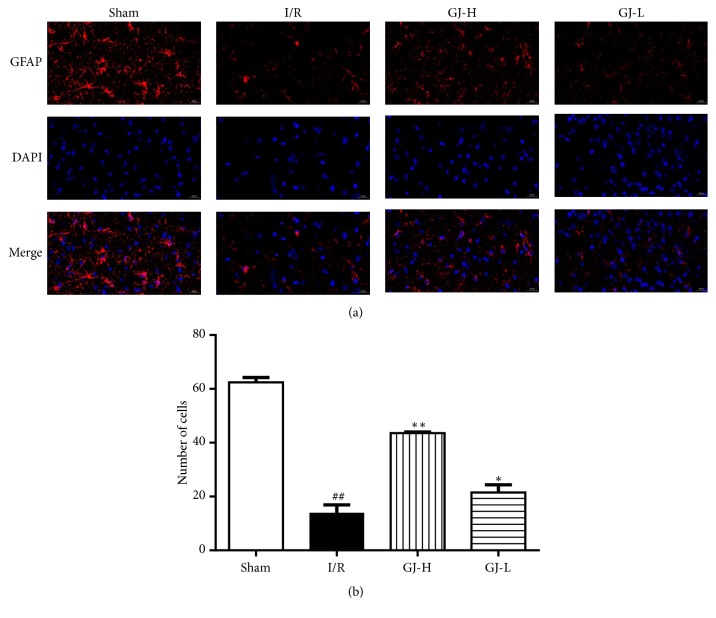
Treatment of different concentration of GJ extracts 7 days prior to MCAO promoted astrocyte proliferation of the ischemic cortex 24 h after reperfusion. (a) Activation of astrocyte was examined with specific antibody against GFAP (red); nuclei were stained with DAPI (blue) (magnification×400). (b) Statistical analysis was shown; data are demonstrated as the mean ± SEM. ^##^*p*<0.01, versus sham group; ^*∗∗*^*p*<0.01, ^*∗*^*p*<0.05 versus I/R group.* Scale bar 20 μm.*

It is reported that GFAP is an astrocyte marker protein [31]; we counted the number of GFAP by immunofluorescence analysis to explore the action mechanisms by which the treatment with GJ extracts may work on the astrocytes during CIR. As shown in Figure 2(a), we observed that most astrocytes in ischemic cortex were died in I/R group induced by MCAO, and the distribution and morphology of GFAP-immunoreactive astrocytes were altered obviously. The numbers of GFAP-immunoreactive astrocytes (13.8±3.1) were statistically decreased compared to sham group (62.4±1.8,* p*<0.01). Nevertheless, the numbers of astrocytes death induced by MCAO were attenuated by pretreatment with GJ extracts at different concentrations. GJ-H group (43.6±0.4) and GJ-L group (21.5±2.9) were markedly increased (*p*<0.01,* p*<0.05, respectively) compared to I/R group (Figure 2(b)).

### 3.3. Effect of GJ Extracts on the Number of Nissl Bodies

**Figure 3:**
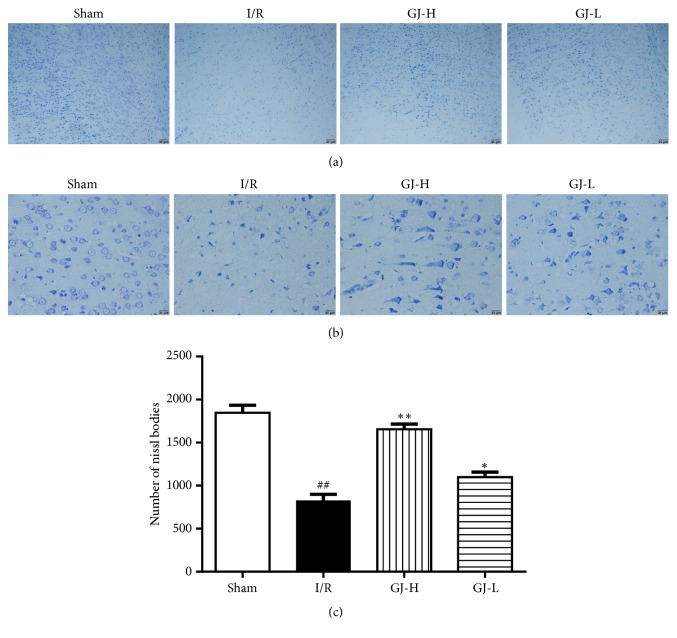
Treatment of different concentration of GJ extracts 7 days prior to MCAO increased the activity of neurons of the ischemic cortex in rat 24 h after reperfusion. Nissl staining was used to identify Nissl bodies at the ischemic cortex after 24 h of reperfusion. (a) Representative images magnified 100 times are shown. (b) Representative images magnified 400 times are shown. (c) Quantitatively analyzed Nissl bodies in the ischemic cortex in each group after 24 h of reperfusion. Data are means ± SEM. ^##^*p*<0.01 versus sham group; ^*∗∗*^*p*<0.01, ^*∗*^*p*<0.05 versus I/R group.* Scale bar 20 μm.*

Nissl staining was evaluated neuronal injury in ischemic cortex after reperfusion for 24 h, and representative Nissl staining of the coronal brain sections was shown in Figures 3(a) and 3(b). In I/R group, many neurons were destroyed and increased intercellular distance indicating injury induced by MCAO; the numbers of Nissl bodies (824.8±74.7) were statistically significant differences compared to sham group (1847.7±86.4) (*p*<0.01). However, those differences were reduced dramatically by GJ extracts therapy at different concentrations. Compared with I/R group, GJ-H group significantly increased the numbers of Nissl bodies (1654.8±60.9,* p*<0.01), while the GJ-L group were upped to (1099.0±58.3) (*p*< 0.05), as shown in Figure 3(c).

### 3.4. Effect of GJ Extracts on Apoptotic Neural Cells

**Figure 4:**
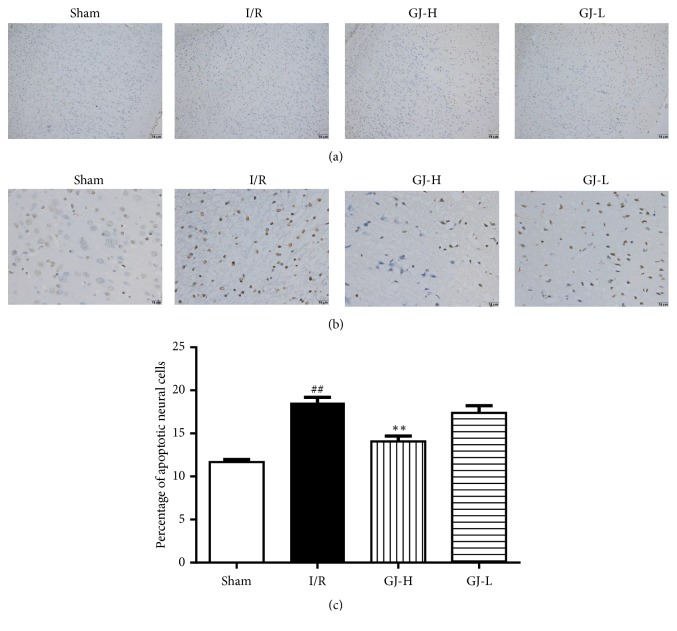
Treatment of different concentration of GJ extracts 7 days prior to MCAO inhibited neural cells apoptosis of the ischemic cortex in rat 24 h after reperfusion. TUNEL staining was used to identify apoptotic neuronal cells in the parietal cortex. (a) Representative images magnified 100 times were shown. (b) Representative images were magnified 400 times. (c) As demonstrated in the bar graphs, the apoptotic index indicates the percentage of TUNEL-positive cells in the ischemic cortex. Data are demonstrated as the mean ± SEM. ^##^*p*<0.01 versus sham-operated group; ^*∗∗*^*p*<0.01 versus I/R group.* Scale bar 10 μm.*

To further examine the effect of pretreatment with GJ extracts on the damage of brain cortex neural cells after CIR, apoptotic neural cells were identified by DNA fragmentation using the TUNEL staining method. The apoptotic index was evaluated by the ratio of the apoptotic cells to the total cell number. Representative photographs were displayed in Figures 4(a) and 4(b). Quantitative analysis displayed in Figure 4(c) revealed that cortex of sham-operated rats was almost negative for DNA fragmentation. Substantial impaired neuronal cells were identified in I/R group (18.54±0.66%), as compared with sham group (11.68±0.28%) which was substantially augment. However, the elevated numbers of TUNEL-positive cells evoked by CIR injury were significantly reduced in the GJ-H group (14.07±0.63%) (*p*<0.01), and we observed that TUNEL-positive cells in GJ-L group (17.38±0.84%) were reduced but had no significant differences compared to I/R group (*p*>0.05).

### 3.5. Effect of GJ Extracts on Bcl-2, Bax, and Cleaved Caspase-3 Expression Assayed by Immunohistochemistry

**Figure 5:**
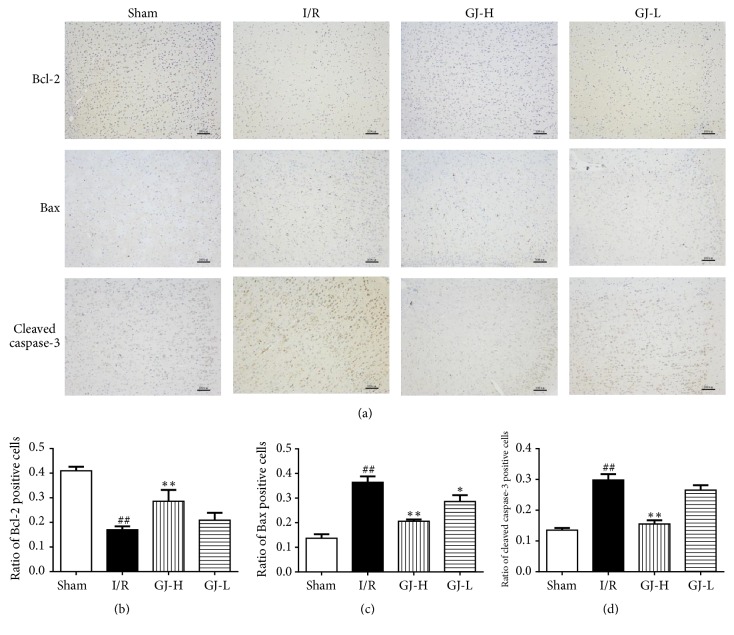
Treatment of different concentration of GJ extracts 7 days prior to MCAO increased Bcl-2 expression and decreased expression of Bax and cleaved caspase-3 by immunohistochemistry. We analyzed the levels of Bcl-2, Bax, and cleaved caspase-3 in brain sections by immunohistochemistry staining. (a) Representative pictures of Bcl-2, Bax, and cleaved caspase-3 positive cells in the tissue. (b, c, and d) As demonstrated in the bar graphs, immunohistochemistry staining results of dates are demonstrated as the mean ± SEM. ^##^*p*<0.01 versus sham group; ^*∗∗*^*p*< 0.01 versus I/R group.* Scale bar 100 μm.*

To determine whether GJ extracts modulate the apoptosis-related proteins, we assessed the effects of GJ extracts on the expression of Bcl-2, Bax, and cleaved caspase-3 by immunohistochemistry staining. Representative immunohistochemistry photographs were shown in Figure 5(a), in I/R group, and Bcl-2 positive cells were markedly increased whereas Bax and cleaved caspase-3 positive cells were decreased significantly compared with sham group (all* p*<0.01). In comparison to I/R group, the Bcl-2 positive cells increased, while Bax and cleaved caspase-3 positive cells decreased in GJ-H group (all* p*<0.01). In GJ-L group, ratio of Bcl-2 and cleaved caspase-3 positive cells had no significant differences compared to I/R group (both* p*>0.05); however, ratio Bax positive cells were significantly decreased compared to I/R group (*p*<0.05).

### 3.6. Effect of GJ Extracts on Bcl-2, Bax, and Cleaved Caspase-3 Expression Assayed by Western Blotting

**Figure 6:**
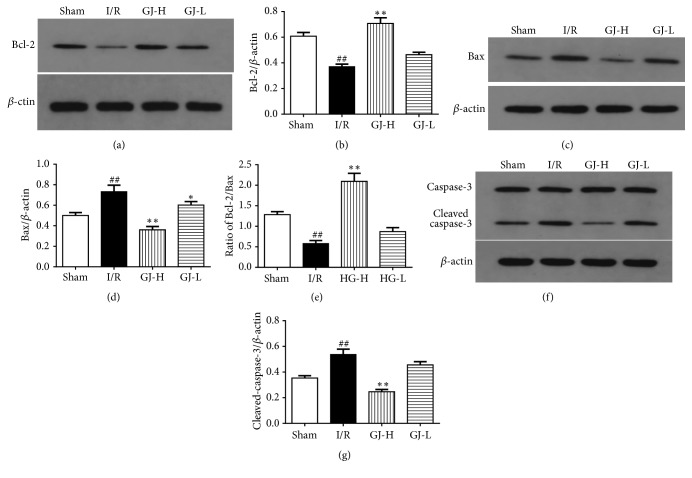
Treatment of different concentration of GJ extracts 7 days prior to MCAO increased Bcl-2 expression and decreased expression of Bax and cleaved caspase-3 by Western blotting. We analyzed the expression levels of Bcl-2, Bax, and cleaved caspase-3 in ischemia cortex. (a, c, and f) The protein band of Bcl-2, Bax, cleaved caspase-3, and corresponding *β*-actin. (b, d, e, and g) As demonstrated in the bar graphs, western blotting results of dates are demonstrated as the mean ± SEM. ^##^*p*<0.01 versus sham group; ^*∗∗*^*p*< 0.01, ^*∗*^*p*<0.05 versus I/R group.

Furthermore, we used western blotting to analyze protein expression of Bcl-2, Bax, and cleaved caspase-3 24 h after reperfusion. Representative photographs for Bcl-2, Bax, and cleaved caspase-3 were exhibited in Figures 6(a), 6(c), and 6(f), respectively. In agreement with results of immunohistochemistry analysis, the protein expression of Bcl-2 was decreased (*p*<0.01); Bax and cleaved caspase-3 were increased in I/R group versus sham group (all* p*<0.01). In comparison to I/R group, the Bcl-2 protein was increased, while Bax and cleaved caspase-3 were decreased in GJ-H group (all* p*<0.01). In GJ-L group, expressions of Bcl-2 and cleaved caspase-3 had no significant differences compared to I/R group (both* p*>0.05); however, Bax was significantly decreased compared to I/R group (*p*<0.05). Bcl-2/Bax ratio in I/R group was significantly decreased compared to sham group (*p*<0.01). The ratio in GJ-H group was significantly increased compared to I/R group (*p*<0.01), but there also had no statistical differences between GJ-L and I/R group (*p*>0.05).

### 3.7. Effect of GJ Extracts on PI3K Expression and Akt Phosphorylation

**Figure 7:**
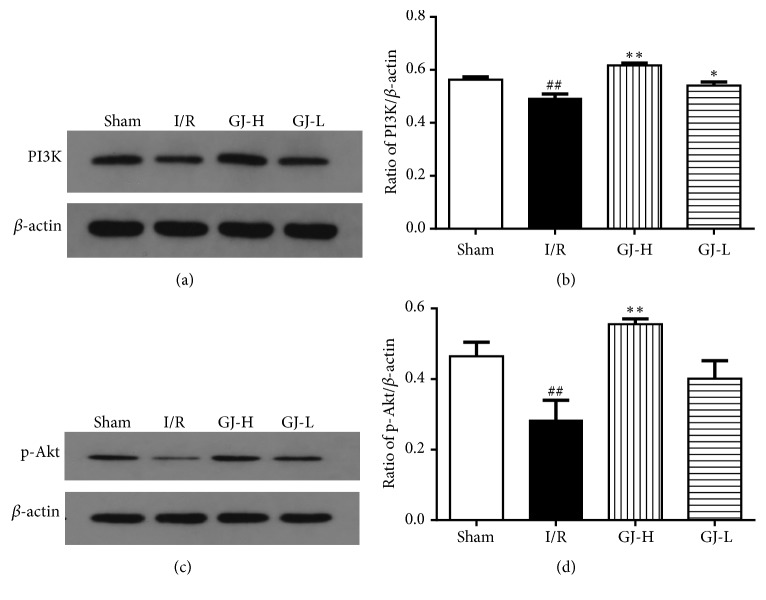
Treatment of different concentration of GJ extracts 7 days prior to MCAO increased the levels of PI3K expression and Akt phosphorylation on cortex neural cells. (a and c) Western blotting bands of PI3K, p-Akt, and corresponding *β*-actin were shown; (b and d) quantitation data of PI3K and p-Akt protein level were shown. ^##^*p*<0.01 versus sham-operated group; ^*∗∗*^*p*< 0.01, ^*∗*^*p*<0.05 versus I/R group.

Moreover, with the aim of exploring the intracellular mechanisms by which the administration with GJ extracts may modulate by the PI3K/Akt signaling pathway during CIR, we evaluated PI3K expression and phosphorylation of Akt levels by western blot analysis at 24 h after reperfusion. As shown in Figure 7, compared to sham group, the PI3K and p-Akt protein expressions were significantly decreased in I/R group (both* p*<0.01), whereas ischemia cerebral cortex levels of PI3K and p-Akt were reduced by administration of GJ extracts. Compared with I/R group, the protein expressions of PI3K and p-Akt were markedly increased in the GJ-H group (both* p*<0.01), and the PI3K protein expression in GJ-L group was also increased when compared with I/R group (*p*<0.05). There was no statistical difference in p-Akt protein expressions between GJ-L and I/R group (*p*>0.05).

## 4. Discussion

As a traditional crude drug* Traditional Chinese Medicine (*TCM), GJ has multiple functions. It has been widely used because of its characteristics of both medicine and food. Accumulating clinical trials and basic research demonstrated that GJ extracts exhibited superior functions in various diseases, such as hyperlipaemia [32], hypertension [33], and cardiac infarction [34–36] as well as cerebral ischemia [37]. Previous researches have showed that the extracts of GJ could promote the supply of oxygen to the brain, reduce cerebral edema and reduce brain injury caused by cerebral ischemia. And it also could alleviate dizziness caused by brain tissue ischemia hypoxia [37]. Liu et al. indicated that GJ extracts could improve learning and memory abilities of ischemia-induced vascular dementia rats/mice, and its mechanism may be related to the promotion of NT-3 and brain derived neurotrophic factor (BDNF) expression, reduction of NF-*κ*B expression, inhibition of the release of inflammatory factors, and the neurons apoptosis [26, 27]. However, the underlying mechanism by which GJ extracts alleviate ischemic stroke damage has not been clearly expounded. Therefore, we investigated whether GJ extracts could decrease apoptosis following CIR injury by activating the PI3K/Akt pathway.

In this study, the neurological function of rats was evaluated by Zea Longa scoring and the recognition of infarct size in ischemic cortex caused by ischemia reperfusion using TTC staining. The results showed that the neurological function was improved, and the area of ischemic infarcts was reduced by GJ extracts preadministration and there were more beneficial effects in the high-dose group* versus* low-dose group (Figure 1), which suggested that a 7-day pretreatment with GJ extracts exerted potent neuroprotection against focal CIR injury in a dose-dependent manner. These results were basically consistent with a previous study by Liu et al. [21]. Since astrocytes and neurons may be the cells in brain tissue which were most affected by CIR injury, here we focused on the cell survival state. We used immunofluorescence staining to detect, an astrocyte-specific marker, and the GFAP expression [38]. And Nissl staining was performed to detect Nissl bodies in the cytoplasm of surviving neurons. In our results, CIR induced the decrease in the number of GFAP positive cells in the cortex (Figure 2), which was partly similar to the findings of Li et al. [39], in which it was indicated that, after 24 h of recirculation, GFAP positive astrocytes disappeared from the ischemic core, while GFAP reactivity increased in the boundary zone, concomitant with transformation of GFAP negative into GFAP positive astrocytes. In our present study, after 1.5 h of ischemia and 24 h of reperfusion, we selected the infarct cortex on the cerebral infarction side as the detection site, which maybe mostly included the core ischemic zone as Li et al. mentioned [40]. Since we were preadministered with GJ extracts, it might be able to control the level of activation of the astrocytes before it is activated by ischemia reperfusion injury. It was observed that the GFAP expression level of the drug group was higher than that of the CIR group in a dose-dependent manner, indicating that the protective effect of GJ extracts was related to the activation and regeneration of astrocytes. In addition, CIR led to the decrease in the number of Nissl bodies, and the number of Nissl bodies could be increased with measurement dependence by GJ extracts (Figure 3). It indicated that GJ extracts would be able to regulate the activity of astrocytes and maintain the normal metabolic level of astrocytes and thereby give certain support to neurons and alleviate neuronal apoptosis, which would further reduce the infarct size and improve neurological function in rats. In a word, the protective effect of GJ extracts was closely related to the maintenance of astrocytes function and the survival of neurons, which was beneficial to ischemic stroke.

Substantial evidence suggested that apoptosis, in addition to necrosis, doses contribute significantly to the cell death subsequent to CIR injury [16, 41, 42]. Thus, we examined the presence of apoptosis in animals using two methods identifying apoptotic cells-TUNEL staining and cleaved caspase-3 immunohistochemistry [43]. Cheung et al. [36] showed that GJ extracts reduced TUNEL-positive myocytes around ischemic area and decreased cleaved caspase-3 positively stained myofibers and vessels. In the present study, we observed that CIR injury evoked increase in the number of TUNEL-positive cells, which was significantly reduced by preconditioned with GJ extracts (Figure 4). The results of cleaved caspase-3 immunohistochemistry indicated that the positive cells of cleaved caspase-3 were decreased by GJ extracts, compared with I/R group. But no significant effects were observed at the lowest dose (1.75 g/kg) of GJ extracts (Figure 5). These observations supported the hypothesis that GJ extracts-mediated neuroprotection was directly correlated to its antiapoptotic mechanism.

In order to characterize the mechanisms underlying apoptosis, we detected the levels of some apoptosis-related proteins. We found that GJ extracts used in advance could increase the expression of Bcl-2 and inhibit Bax expression after CIR injury. In other words, the ratio of Bcl-2/Bax was increased by GJ extracts (Figures 6(a), 6(b), 6(c), 6(d), and 6(e)). The ratio of Bcl-2/Bax appeared to determine whether the cells survived or dead under apoptotic stimulus [44]. Studies indicated that Bcl-2 and Bax play an important role in cell apoptosis, they belong to the Bcl-2 proteins family. Among them, Bcl-2 facilitates cell survival while Bax accelerates apoptosis [23, 45, 46]. The antiapoptosis effect may cause the restoration of blood supply or the initiation of the intracerebral revascularization. The effect of GJ extracts was also observed in the ischemic myocardium by Gu et al. [47], who in their study has demonstrated that GJ extracts could promote the expression of angiogenic factors, restore early revascularization, and improve blood supply to ischemic myocardium. At the same time, it could also induce the expression of antiapoptotic protein Bcl-2 in infarcted hearts and inhibit the apoptosis of cardiomyocytes. Furthermore, caspase-3, an executioner in the family of cysteine proteases, had generally been considered as a terminal step of apoptotic cell death in the acute phase of stroke. Caspase-3 could promote the apoptotic cell death and was detected by investigating the protein expression of cleaved caspase-3 [48]. Our data exhibited that the protein expression of cleaved caspase-3 in rat cerebral cortex was significantly increased after ischemia and reperfusion treatment, and these status could be significantly alleviated by the GJ extracts pretreatment (Figures 6(f) and 6(g)).

As an important signaling pathway in neuroprotection and antiapoptosis, PI3K/Akt pathway was inhibited after ischemia and reperfusion, which activated the pro-apoptotic pathway [25, 49]. Our results indicated that PI3K/Akt pathway was related to the neuroprotective effect of GJ extracts on CIR. The data of Western blotting confirmed that the level of PI3K was significantly decreased in the ischemic cortex induced by CIR, while the expression of PI3K was markedly improved by GJ extracts pretreatment and the effect of GJ-H group was better than GJ-L group (Figures 7(a) and 7(b)). In the central nervous system, the suppression of Akt activity has been associated with CIR-induced neuronal death [48]. Previous works have shown that phosphorylation of Akt (Ser473) was necessary for Akt to activate fully [40, 49]. Therefore, the degree of activation of Akt was indicated by detection of p-Akt (Ser473) protein, it was significantly reduced in IR group, and this phenomenon was attenuated by high-dose GJ extracts pretreatment. Although low-dose GJ extracts also increased the expression of p-Akt, the results did not show statistically significant (Figures 7(c) and 7(d)). It showed that GJ extracts could exert its function of antiapoptosis by upregulating Akt phosphorylation. In addition, a research has found that GJ extracts could induced the protein Akt and Bcl-2 expression and inhibited cardiomyocytes death of in the infarcted hearts, which may conducive to the increased livability of the viable myocytes at risk after infarction [36]. According to the comprehensive description as above, GJ extracts could alleviate neuronal apoptosis after focal CIR injury, prevent further ischemic injury, and reduce the infarct size, these effects were closely related to the activation of PI3K/Akt signaling pathway.

## 5. Conclusion

**Figure 8:**
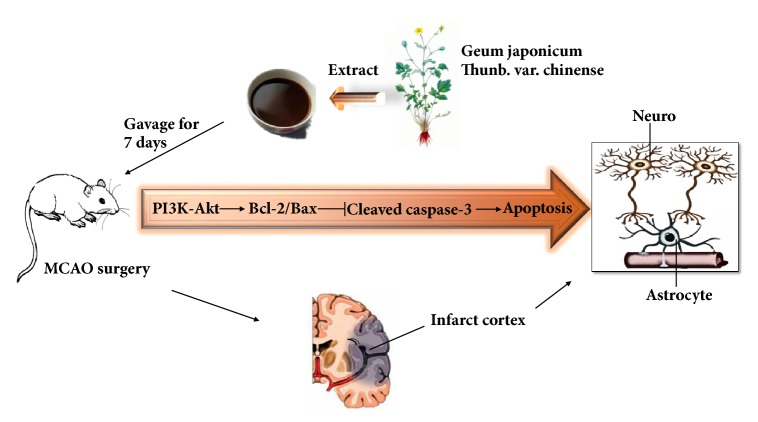
Proposed scheme of GJ extracts suppressed CIR-induced apoptosis via PI3K/Akt signaling pathway. GJ extracts pretreatment significantly improved the neurological deficits, decreased infarct volume, downregulated the level of cleaved caspase-3, and upregulated the levels of Bcl-2/ Bax, which could be caused by promoting PI3K expression and phosphorylation with Akt.

In the current study, we demonstrated that preconditioning with GJ extracts prevents focal CIR injury in SD rats, and the underlying mechanisms might be related to reducing apoptosis via modulating PI3K/Akt signaling pathway and regulating the balance of apoptosis-related proteins Bcl-2 and Bax, furthermore increasing the survival of neurons and upregulating the activity of astrocytes, and reducing neurological deficit scores and reducing infarct volume (Figure 8). These results provided new insights into the action mechanism of GJ extracts, which may improve CIR injury by inhibiting apoptosis. It indicated that GJ extracts may be used as a preventative or complementary medicine against ischemic stroke and other ischemic cerebral diseases. However, our experiment in this study was a preliminary exploration for the pharmacological effects of GJ, and there are some limitations. For example, it is unclear which chemical components play a major role in the prevention and treatment of CIR injury, and further separation and purification work is needed in our follow-up research. On the other hand, although GJ has been used as a TCM for hundreds of years in the folk, no side effect or toxicity has been observed or reported; in the future more stringent toxicity experiment should be carried out. Finally, the lack of positive or negative controls in the experiment should be remedied in future study.

## Data Availability

The data used to support the findings of this study are available from the corresponding author upon request.
